# Supplementary material for: Collection of biospecimens from the inspiration4 mission establishes the standards for the space omics and medical atlas (SOMA)
Source: Nat Commun. 2024 Jun 11;15:4964. doi: 10.1038/s41467-024-48806-z (PMC11166662; doi:10.1038/s41467-024-48806-z)
Supplement: Supplementary file 1 — Supplementary Information [file 41467_2024_48806_MOESM1_ESM.pdf]

Supplementary Information Directory

Collection of Biospecimens from the Inspiration4 Mission Establishes the Standards for the Space Omics and Medical Atlas (SOMA).

Eliah G. Overbey, Krista Ryon, JangKeun Kim, Braden T. Tierney, Remi Klotz, Veronica Ortiz, Sean Mullane, Julian C. Schmidt, Matthew MacKay, Namita Damle, Deena Najjar, Irina Matei, Laura Patras, J. Sebastian Garcia Medina, Ashley Kleinman, Jeremy Wain Hirschberg, Jacqueline Proszynski, S. Anand Narayanan, Caleb M. Schmidt, Evan E. Afshin, Lucinda Innes, Mateo Mejia Saldarriaga, Michael A. Schmidt, Richard D. Granstein, Bader Shirah, Min Yu, David Lyden, Jaime Mateus, Christopher E. Mason

Supplementary Table 1: Prior Biospecimen Collections from Astronauts. **2**
Supplementary Table 2: Aliquot, Kit, and Swab Counts. **8**
Supplementary Table 3: Blood Derivative Allocations. **10**
Supplementary Table 4: Blood Derivative Aliquot Parameters. **11**
Supplementary Fig 1: Field Processing of Blood Tubes. **12**

Supplementary Table 1: Prior Biospecimen Collections from Astronauts.

Listed studies are limited to the past decade. Abbreviations: L- = before launch. FD = flight day. R+ = after return. PBMC = peripheral blood mononuclear cell.

Sample(s)	Measure(s)	Number of Subjects (n)	Duration Range (days)	Collection Time points	Study (citation)
Salivary					
Saliva	Salivary Microbiome	10	60-270	L-180, L-90<br>FD 1-2 months<br>FD 2-4 months<br>FD (R-10)<br>R+0, R+30, R+60, R+180	[1]
Blood (Single Component)					
Blood (Plasma)	mtDNA, Long Non-coding RNA, Exosomes	3-14	5-13	L-10, R-0, R+3	[2,3]
Blood (Plasma)	Cytokines	28	~180	L-180, L-45, L-10, FD15,30,60,120,180; R+0, R+30	[4]
Blood (Plasma)	Proteomics	13-18	169-199	L-30, R+0, R+7	[5–8]
Blood (Plasma)	sRNAseq (miRNA from sEV)	14	12 (median)	L-10, R+0, R+3	[9]
Blood (PBMCs)	Peripheral Leukocyte Distribution, T-cell Function, Virus-specific Immunity, and Mitogen-stimulated Cytokine Production Profiles	23	<60 days (n=2), >100 days (n=5), 180 days (n=16)	L-180, L-45, FD14, FD 2-4 mn, FD6 mn, R+0, R + 30	[10]

Blood (Serum)	Metabolic Profile	51	120-180	L-45, L-10, FD15, FD30, FD60, FD120, FD180, R+0, R+30	[11]
Blood	Natriuretic Peptide, Creatinine, Aldosterone, Sodium	8	Long Duration	Not specified	[12]
Blood (Whole Blood)	Transcriptome	6	10-13	L-10, R+0 (2-3 hour after return)	[13]
Blood (Whole Blood)	Hematology	31	Up to 180	L-180, L-45; FD-14, FD60-FD120, FD180, R+0, R+30	[14]
Blood (Multiple Components)					
Blood (Plasma, PBMCs)	snoRNA Expression Levels	n=5 (plasma), n=6 (PBMCs)	14 (median)	L-10, R+3	[15]
Blood (Serum, Whole Blood)	Hematology<br>Hemolysis (Serum Iron)<br>Heme Degradation	14	167 ± 31 days (mean±sd)	L-100, FD5, FD11, FD64, FD157, R+4, R+14, R+41, R+184< R+365	[16]
Blood (Whole Blood, Plasma)	Immunophenotyping, NK Cell cytotoxicity and conjugation, Degranulation, Plasma stimulation	9	180 to 340 days	L-180, L-60< FD90, FD180 (n=1), R-1, R+0, R+18, R+33, R+66	[17]
Blood (PBMCs, Plasma, Whole Blood)	Leukocyte Distribution, T cell Blastogenesis, and Cytokine Production Profiles	19	10-15	L-180, L-10, in-flight (R-1), R+0, R+14	[18]
Blood and Saliva					
Blood (Plasma), Saliva	Cytokines	13	140-290	L-180, L-45, L-10, FD15, FD30, FD60, FD120, FD180, R+0, and R+30	[19]

Blood (Plasma, PBMCs, Whole Blood), Saliva	Immune Cell Counts, Cortisol	9	162	L-25, FD90, FD150, R+1, R+7, R+30	[20]
Blood and Core Body Temperature					
Core Body Temperature (CBT), Blood (Whole Blood)	CBT, IL-1ra	11	180	CBT: L-90, FD15, FD45, FD75, FD105, FD135, FD165, R+1, R+10, R+30<br>Blood: L-180, L-45, L-10, FD15, FD30, FD60, FD120, FD180, R+0, R+30	[21]
Blood and Urine					
Blood (Plasma, PBMCs), Urine	Thymopoiesis	16	184 (median)	Regular Intervals (preflight, return, postflight)	[22]
Blood (Serum, Plasma, Whole Blood), Urine	Iron Status	23	50-247	L-180, L-45, L-10, FD15, FD30, FD60, FD120, FD180, R+0, R+30	[23]
Blood, Urine	Bone Loss and Kidney Stone Risk	42	49-215	10-131 days before flight and after flight (R+0, R+1 and R+2)	[24]
Blood (Serum), Urine	Bone Metabolism and Renal Stone Risk	23	120-180	L-180, L-45, L-10, FD15, FD30, FD60, FD120, FD180	[25]
Blood (Serum), Urine	Bone Metabolism	17	160 +/-20	L-180, L-45, FD15, FD30, FD60, FD120, FD180	[26]
Multiple Samples (3) – Blood, Urine, and Saliva					

Blood (Plasma, Whole Blood), Saliva, Urine	B Cell Phenotyping Ig Analyses	Integral Immune Study (n=15)<br><br>Salivary Markers Study (n=8)	180	Salivary:<br>Plasma: L-180, L-45, FD10, FD90, FD180/R-1, R+0, R+30<br>Salivary Marker Study: L-180, L-60, FD-10, FD-90, FD-180/R-1, R+0, R+18, R+33, and R+66	[27]
Saliva, Blood (Plasma), Urine	Salivary Biomarkers, Stress biomarkers	15	180	L-180, L-60, FD10, FD90, R-1, R+0, R+18, R+33, R+66	[28]
Blood, Urine, Saliva	Antiviral Antibodies and Viral Load	17	12-16	Blood, Urine: L-180, L-10, R+0, R+14<br>Saliva Dry: L-180, L-10, FD1, FD11, R+1, R+14<br>Saliva Liquid: L-180, L-10, FD1,FD3,FD5,FD7,FD9,FD11 R+0, R+2, R+4, R+6, R+8, R+10, R+12, R+14	[29]
Multiple Samples (3) - Blood, Urine, Imaging					
Blood (Serum) Urine, Ultrasound	Arterial Structure and Function	13	126-340	L-180, L-60, FD15, FD60, FD160, R+5	[30]
Blood (Serum), Urine, quantitative CT	Bone Metabolism, Bone Density, Bone Strength	17	105-210 (mean: 170 days)	Blood/Urine: L-180, L-45, FD15, FD30, FD60, FD120, FD180, R+0	[31]
Swabs & Multiple Samples (3+)					
ISS Section Swab	Metagenomics, Physiological Characterization of Microbes	n/a	n/a	3 timepoints (session A, B, and C)	[32]
Body Swabs, Saliva	Metagenomics	4	Not reported.	L-180, L-45; FD-14, FD60-FD120, FD180, R+0, R+30, R+180	[33]

Saliva, Body Swabs, 8 Environmental Locations	Microbiome	1	135	Before, During, After Spaceflight (L-180, L-90; FD60, FD97, FD126, R+1, R+30, R+180)	[34]
Blood (Plasma), Microbiome Swabs, Stool, Saliva, Environmental Swabs	Metagenomics, Cytokine	9	180 - 360	L-240, L-160, L-90, L-60, FD7, FD90, FD126, R+0/3, R+30, R+60, R+180	[35]
Blood (Serum, Plasma), Urine, Saliva, Skin Swab	Antiviral antibodies and viral load (DNA) were measured for Epstein-Barr virus (EBV), varicella-zoster virus (VZV), and cytomegalovirus (CM)	17	12-16	Saliva: L-180, L-10, every other day during flight, and every other day post flight until R+14<br>Blood/Urine: L-180, L-10, R+0, R+14	[36]
Blood (Serum), Urine, Epithelial Cells (Sublingual Mucosa)	Magnesium	43	120-180	Serum/Urine: L-180, L-45, FD15, FD30, FD60, FD120, FD180, R+0, R+30<br>Tissue: L-180, L-45, R+0, R+30	[37]
Blood, Urine, Human Hair, Human T cells	Multi-omics	59	120-180	L-180, L-45, FD15, FD30, FD60, FD120, FD180, R+0, R+30	[38]
NASA Twins Study Analysis					
Stool, Saliva, Skin, Urine, Blood (Plasma, PBMCs, Lymphocyte-depleted cells)	Metabolomics, Proteomics, Cognition, Microbiome, Telomeres, Epigenomics, Biochemical Profile, Gene Expression, Integrative Omics, Immunome	2	340	Before, during, and after spaceflight	[39]

Blood	Clonal Hematopoiesis Panel, Whole Genome Sequencing, RNA-seq	2	340	Before, during, and after spaceflight	[40]
Blood	Uremic Toxin *p*-Cresol	2	340	Before, During, and After Spaceflight	[41]
Blood (Plasma)	Cell-free DNA, Exosome	2	340	Before, during, and after spaceflight (12 timepoints from twin on earth and 11 from twin in space)	[42]
Blood (WBCs, PBMCs, Lymphocyte-depleted Fraction)	Multi-omic, Untargeted RNA-seq	2	340	Before, During, and After Spaceflight	[43]
Blood (PBMCs, Lymphocyte-depleted Fraction)	Circulating miRNA	2	340	Before, during, and after flight	[44]
Blood (Plasma PBMCs), Urine	Telomere Length Plasma Cytokine	3	340, 180	Blood: L-270, L-180, L-60, FD45, FD90, FD140, FD260, R+1, R+180, R+270<br>Urine: L-180, L-45, FD15, FD240, FD330, R+1, R+60<br>Biochemistry:L-80, L-45, FD15, FD30, FD60, FD120, FD180, R+0, R+30	[45]
Blood (PBMCs, Lymphocyte-depleted Fraction)	Circulating miRNA	2	340	Before, during, and after flight	[44]
Blood (Plasma, PBMCs, Lymphocyte-depleted Fraction), Urine	Multi-omic, Single-Cell, Biochemical Measures	2	340	Before, during, and after spaceflight	[46]

Supplementary Table 2: **Aliquot, Kit, and Swab Counts.**

Total number of samples returned to WCM from field processing locations over the course of the study.

Sample Type	Total Sample Count Across Mission
Blood Derivatives	
Plasma	510 Aliquots
Serum	180 Aliquots
PBMCs	306 Aliquots
Red Blood Cell (RBC) Pellets	98 Aliquots
PAXgene Preserved RNA Tubes	29 Tubes
Dried Blood Spots (DBSs)	140 Spots
Saliva	
OMNIgene•ORAL Saliva Kit	24 Collection Kits
Crude Saliva	201 Aliquots
Urine	
Urine with Zymo Urine Conditioning Buffer - 1mL Aliquots	314 Aliquots
Urine with Zymo Urine Conditioning Buffer - Conical Tubes	87 Tubes
Crude Urine - 1mL Aliquots	330 Aliquots
Crude Urine - Conical Tubes	114 Tubes
Stool	
OMNIgene•GUT Microbiome Preservation	8 Collection Kits
OMNImet•GUT Metabolome Preservation	8 Collection Kits
Skin Swabs	
Isohelix Swabs in Zymo DNA/RNA Shield	359 Swabs
Skin Biopsy	
Formalin-Fixed Tissue	8 Specimens

Snap-Frozen Tissue	8 Specimens
Environmental Swabs and HEPA Filter	
Isohelix Swabs in Zymo DNA/RNA Shield	40 Swabs
HEPA Filter	147 Sections
**Total:**	**2,911 Samples**

Supplementary Table 3: Blood Derivative Allocations.

Samples types collected, their tube type of origin, and assay allocation. Samples collected in excess were biobanked to enable additional experiments as new assays are developed. Abbreviations: PBMC = peripheral blood mononuclear cell. gDNA = genomic DNA. cfDNA = cell-free DNA. GEX = gene expression. BCR = B-cell repertoire. TCR = T-cell repertoire. EVPs = extracellular vesicles and particles.

Sample Type	Tube Source	Closure Color	Assay Allocation(s)
Whole Blood	BD PAXgene® Blood RNA Tube (bRNA)	Red	Total RNA Extraction
Plasma	BD Vacutainer® Mononuclear Cell Preparation Tube (CPT)	Red/Green	Proteomics, Metabolomics; Biobanking
PBMCs	BD Vacutainer® Mononuclear Cell Preparation Tube (CPT)	Red/Green	Biobanking
Red Blood Cell Pellet	BD Vacutainer® Mononuclear Cell Preparation Tube (CPT)	Red/Green	gDNA; Biobanking
Serum	BD Vacutainer® Serum Separator Tubes (SST)	Red/Grey	Immune and Cardiovascular Disease Panel, Metabolic Panel; Biobanking
Red Blood Cell Pellet	BD Vacutainer® Serum Separator Tubes (SST)	Red/Grey	gDNA; Biobanking
Plasma	Streck Cell-Free DNA BCT® (cfDNA BCT)	Tan/Black	cfDNA; Biobanking
Red Blood Cell Pellet	Streck Cell-Free DNA BCT® (cfDNA BCT)	Tan/Black	gDNA; Biobanking
PBMCs	BD Vacutainer® K2 EDTA	Lavender/C	Single-Cell Multiome GEX+ATAC and BCR/TCR Immune Profiling
Plasma	BD Vacutainer® K2 EDTA	Lavender/C	EVPs
Whole Blood	BD Vacutainer® K2 EDTA	Lavender/C	Complete Blood Count

Supplementary Table 4: Blood Derivative Aliquot Parameters.

Plasma, serum, and PBMCs aliquots were created for downstream assays that only require a portion of the total sample collected in order to minimize freeze-thaw cycles. Abbreviations: PBMC = peripheral blood mononuclear cell. cfDNA BCT = cell-free DNA blood collection tube. CPT = cell processing tube. SST = serum separator tube.

Sample Type	Tube Source	Aliquot Sizes	Freezing Condition at Field Laboratory	Freezing Condition at Central Lab (WCM)
Plasma	cfDNA BCT	500 uL	-80°C Freezer	-80°C Freezer
Plasma	CPT	500 uL	-80°C Freezer	-80°C Freezer
Serum	SST	500 uL	-80°C Freezer	-80°C Freezer
PBMCs	CPT	⅙ tube yield	-80°C Freezer (slow freeze)	-196°C Liquid Nitrogen

Supplementary Fig 1: Field Processing of Blood Tubes.

Some assays were performed immediately on fresh samples (orange boxes), while all other assays were performed on frozen samples mailed to our central laboratory facilities at Weill Cornell Medicine. The comprehensive metabolic panel and complete blood count performed by Quest Diagnostics used fresh samples of serum and whole blood respectively. The 10X Genomics single-cell protocols were also performed on fresh PBMCs isolated from K2 EDTA tubes. One K2 EDTA tube was immediately mailed on ice to a collaborator's lab for exosome isolation. All remaining assays were performed on biobanked aliquots of plasma, PBMCs, red blood cell pellets (RBCs), and serum.

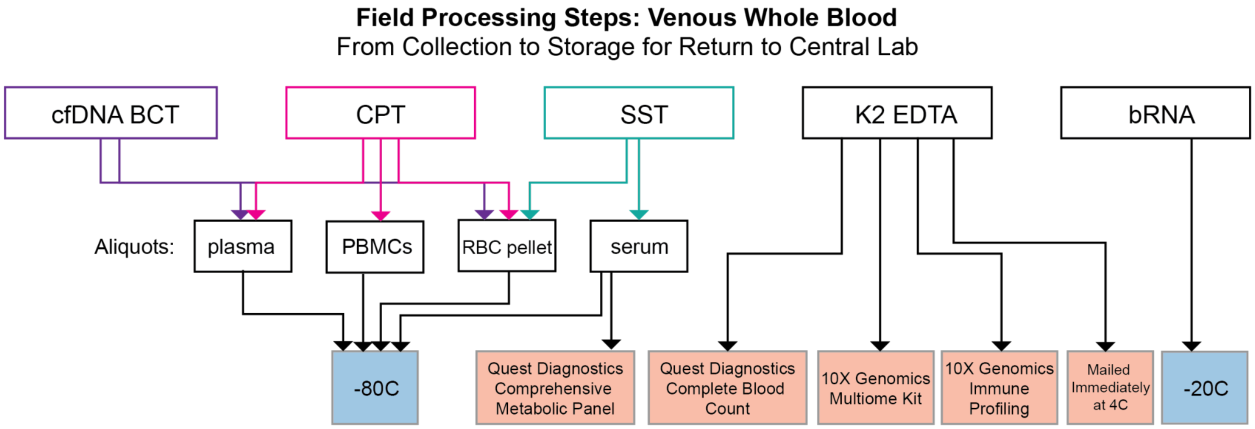

References

1. Urbaniak, C. *et al.* The influence of spaceflight on the astronaut salivary microbiome and the search for a microbiome biomarker for viral reactivation. *Microbiome* **8**, 56 (2020).
2. Bisserier, M. *et al.* Cell-Free Mitochondrial DNA as a Potential Biomarker for Astronauts' Health. *J. Am. Heart Assoc.* **10**, e022055 (2021).
3. Bisserier, M. *et al.* Emerging Role of Exosomal Long Non-coding RNAs in Spaceflight-Associated Risks in Astronauts. *Front. Genet.* **12**, 812188 (2021).
4. Crucian, B. E. *et al.* Plasma Cytokine Concentrations Indicate That In Vivo Hormonal Regulation of Immunity Is Altered During Long-Duration Spaceflight. *J. Interferon Cytokine Res.* **34**, 778–786 (2014).
5. Brzhozovskiy, A. G. *et al.* The Effects of Spaceflight Factors on the Human Plasma Proteome, Including Both Real Space Missions and Ground-Based Experiments. *Int. J. Mol. Sci.* **20**, 3194 (2019).
6. Kashirina, D. N. *et al.* The molecular mechanisms driving physiological changes after long duration space flights revealed by quantitative analysis of human blood proteins. *BMC Medical Genomics* vol. 12 Preprint at https://doi.org/10.1186/s12920-019-0490-y (2019).
7. Larina, I. M. *et al.* Protein expression changes caused by spaceflight as measured for 18 Russian cosmonauts. *Sci. Rep.* **7**, 8142 (2017).
8. Kashirina, D. N., Pastushkova, L. K. & Percy, A. J. Changes in the plasma protein composition in cosmonauts after space flight and its significance for endothelial functions. *Hum. Physiol.* (2019) doi:10.1134/S0362119719010092.
9. Goukassian, D. & Arakelyan, A. Space flight associated changes in astronauts' plasma-derived small extracellular vesicle microRNA: Biomarker identification. *Clinical and* (2022).
10. Crucian, B. *et al.* Alterations in adaptive immunity persist during long-duration spaceflight. *NPJ Microgravity* **1**, 15013 (2015).
11. Stroud, J. E. *et al.* Longitudinal metabolomic profiles reveal sex-specific adjustments to long-duration spaceflight and return to Earth. *Cell. Mol. Life Sci.* **79**, 578 (2022).
12. Frings-Meuthen, P. *et al.* Natriuretic Peptide Resetting in Astronauts. *Circulation* **141**, 1593–1595 (2020).
13. Barrila, J. *et al.* Spaceflight modulates gene expression in the whole blood of astronauts. NPJ Microgravity 2: 16039. Preprint at (2016).
14. Kunz, H. *et al.* Alterations in hematologic indices during long-duration spaceflight. *BMC Hematol* **17**, 12 (2017).
15. Rai, A. K. *et al.* Spaceflight-Associated Changes of snoRNAs in Peripheral Blood Mononuclear Cells and Plasma Exosomes—A Pilot Study. *Frontiers in Cardiovascular Medicine* **9**, (2022).
16. Trudel, G., Shahin, N., Ramsay, T., Laneuville, O. & Louati, H. Hemolysis contributes to anemia during long-duration space flight. *Nat. Med.* **28**, 59–62 (2022).
17. Bigley, A. B. *et al.* NK cell function is impaired during long-duration spaceflight. *J. Appl. Physiol.* **126**, 842–853 (2019).
18. Crucian, B. *et al.* Immune system dysregulation occurs during short duration spaceflight on board the space shuttle. *J. Clin. Immunol.* **33**, 456–465 (2013).
19. Krieger, S. S. *et al.* Alterations in Saliva and Plasma Cytokine Concentrations During Long-Duration Spaceflight. *Front. Immunol.* **12**, 725748 (2021).
20. Buchheim, J.-I. *et al.* Stress Related Shift Toward Inflammaging in Cosmonauts After Long-Duration Space Flight. *Front. Physiol.* **10**, 85 (2019).
21. Stahn, A. C. *et al.* Increased core body temperature in astronauts during long-duration space missions. *Sci. Rep.* **7**, 16180 (2017).
22. Benjamin, C. L. *et al.* Decreases in thymopoiesis of astronauts returning from space flight. *JCI Insight* **1**, e88787 (2016).

23. Zwart, S. R., Morgan, J. L. L. & Smith, S. M. Iron status and its relations with oxidative damage and bone loss during long-duration space flight on the International Space Station. *Am. J. Clin. Nutr.* **98**, 217–223 (2013).
24. Smith, S. M. *et al.* Men and Women in Space: Bone Loss and Kidney Stone Risk After Long-Duration Spaceflight. *Journal of Bone and Mineral Research* vol. 29 1639–1645 Preprint at https://doi.org/10.1002/jbmr.2185 (2014).
25. Smith, S. M. *et al.* Bone metabolism and renal stone risk during International Space Station missions. *Bone* **81**, 712–720 (2015).
26. Zwart, S. R. *et al.* Dietary acid load and bone turnover during long-duration spaceflight and bed rest. *Am. J. Clin. Nutr.* **107**, 834–844 (2018).
27. Spielmann, G. *et al.* B cell homeostasis is maintained during long-duration spaceflight. *J. Appl. Physiol.* **126**, 469–476 (2019).
28. Agha, N. H. *et al.* Salivary antimicrobial proteins and stress biomarkers are elevated during a 6-month mission to the International Space Station. *J. Appl. Physiol.* **128**, 264–275 (2020).
29. Mehta, S. K. *et al.* Multiple latent viruses reactivate in astronauts during Space Shuttle missions. *Brain Behav. Immun.* **41**, 210–217 (2014).
30. Lee, S. M. C. *et al.* Arterial structure and function during and after long-duration spaceflight. *J. Appl. Physiol.* **129**, 108–123 (2020).
31. Gabel, L. *et al.* Pre-flight exercise and bone metabolism predict unloading-induced bone loss due to spaceflight. *Br. J. Sports Med.* **56**, 196–203 (2022).
32. Mora, M. *et al.* Space Station conditions are selective but do not alter microbial characteristics relevant to human health. *Nat. Commun.* **10**, 3990 (2019).
33. Morrison, M. D. *et al.* Investigation of Spaceflight Induced Changes to Astronaut Microbiomes. *Front. Microbiol.* **12**, 659179 (2021).
34. Avila-Herrera, A. *et al.* Crewmember microbiome may influence microbial composition of ISS habitable surfaces. *PLoS One* **15**, e0231838 (2020).
35. Voorhies, A. A. *et al.* Study of the impact of long-duration space missions at the International Space Station on the astronaut microbiome. *Scientific Reports* vol. 9 Preprint at https://doi.org/10.1038/s41598-019-46303-8 (2019).
36. Mehta, S. K. *et al.* Dermatitis during Spaceflight Associated with HSV-1 Reactivation. *Viruses* **14**, (2022).
37. Smith, S. M. & Zwart, S. R. Magnesium and Space Flight. *Nutrients* **7**, 10209–10222 (2015).
38. da Silveira, W. A. *et al.* Comprehensive Multi-omics Analysis Reveals Mitochondrial Stress as a Central Biological Hub for Spaceflight Impact. *Cell* **183**, 1185–1201.e20 (2020).
39. Garrett-Bakelman, F. E. *et al.* The NASA Twins Study: A multidimensional analysis of a year-long human spaceflight. *Science* **364**, (2019).
40. Mencia-Trinchant, N. *et al.* Clonal hematopoiesis before, during, and after human spaceflight. *Cell Rep.* **34**, 108740 (2021).
41. Schmidt, M. A., Meydan, C., Schmidt, C. M., Afshinnekoo, E. & Mason, C. E. Elevation of gut-derived p-cresol during spaceflight and its effect on drug metabolism and performance in astronauts. *bioRxiv* (2020) doi:10.1101/2020.11.10.374645.
42. Bezdan, D. *et al.* Cell-free DNA (cfDNA) and Exosome Profiling from a Year-Long Human Spaceflight Reveals Circulating Biomarkers. *iScience* **23**, 101844 (2020).
43. Schmidt, M. A., Meydan, C., Schmidt, C. M., Afshinnekoo, E. & Mason, C. E. The NASA Twins Study: The Effect of One Year in Space on Long-Chain Fatty Acid Desaturases and Elongases. *Lifestyle Genom* **13**, 107–121 (2020).
44. Malkani, S. *et al.* Circulating miRNA Spaceflight Signature Reveals Targets for Countermeasure Development. *Cell Rep.* **33**, 108448 (2020).
45. Luxton, J. J. *et al.* Temporal Telomere and DNA Damage Responses in the Space

Radiation Environment. *Cell Rep.* (2020) doi:10.2139/ssrn.3646569.
46. Gertz, M. L. *et al.* Multi-omic, Single-Cell, and Biochemical Profiles of Astronauts Guide Pharmacological Strategies for Returning to Gravity. *Cell Rep.* **33**, 108429 (2020).
