# Supplementary material for: Collection of biospecimens from the inspiration4 mission establishes the standards for the space omics and medical atlas (SOMA)
Source: Nat Commun. 2024 Jun 11;15:4964. doi: 10.1038/s41467-024-48806-z (PMC11166662; doi:10.1038/s41467-024-48806-z)
Supplement: Supplementary file 3 — Reporting Summary [file 41467_2024_48806_MOESM3_ESM.pdf]

nature portfolio

Corresponding author(s): Christopher E Mason

Last updated by author(s): Apr 20, 2024

Reporting Summary

Nature Portfolio wishes to improve the reproducibility of the work that we publish. This form provides structure for consistency and transparency in reporting. For further information on Nature Portfolio policies, see our Editorial Policies and the Editorial Policy Checklist.

Statistics

For all statistical analyses, confirm that the following items are present in the figure legend, table legend, main text, or Methods section.

n/a	Confirmed	
☐	☒	The exact sample size (*n*) for each experimental group/condition, given as a discrete number and unit of measurement
☐	☒	A statement on whether measurements were taken from distinct samples or whether the same sample was measured repeatedly
☒	☐	The statistical test(s) used AND whether they are one- or two-sided<br>*Only common tests should be described solely by name; describe more complex techniques in the Methods section.*
☒	☐	A description of all covariates tested
☒	☐	A description of any assumptions or corrections, such as tests of normality and adjustment for multiple comparisons
☐	☒	A full description of the statistical parameters including central tendency (e.g. means) or other basic estimates (e.g. regression coefficient) AND variation (e.g. standard deviation) or associated estimates of uncertainty (e.g. confidence intervals)
☒	☐	For null hypothesis testing, the test statistic (e.g. *F*, *t*, *r*) with confidence intervals, effect sizes, degrees of freedom and *P* value noted<br>*Give P values as exact values whenever suitable.*
☒	☐	For Bayesian analysis, information on the choice of priors and Markov chain Monte Carlo settings
☒	☐	For hierarchical and complex designs, identification of the appropriate level for tests and full reporting of outcomes
☒	☐	Estimates of effect sizes (e.g. Cohen's *d*, Pearson's *r*), indicating how they were calculated

Our web collection on statistics for biologists contains articles on many of the points above.

Software and code

Policy information about availability of computer code

Data collection	Software was run the measure DNA and RNA nucleic acid concentrations on the Qubit 4 Fluorometer and protein concentrations on the Promega GloMax Plate Reader running manufacturer's software.
Data analysis	N/A

For manuscripts utilizing custom algorithms or software that are central to the research but not yet described in published literature, software must be made available to editors and reviewers. We strongly encourage code deposition in a community repository (e.g. GitHub). See the Nature Portfolio guidelines for submitting code & software for further information.

Data

Policy information about availability of data

All manuscripts must include a data availability statement. This statement should provide the following information, where applicable:

- Accession codes, unique identifiers, or web links for publicly available datasets
- A description of any restrictions on data availability
- For clinical datasets or third party data, please ensure that the statement adheres to our policy

Images of the Inspiration4 and Dragon Capsule are available from the SpaceX Flickr Account (https://www.flickr.com/people/spacex/). This was the source image data used to label the Dragon swab location in Fig 9.

Research involving human participants, their data, or biological material

Policy information about studies with human participants or human data. See also policy information about sex, gender (identity/presentation), and sexual orientation and race, ethnicity and racism.

Reporting on sex and gender	The crew included two men and two women.
Reporting on race, ethnicity, or other socially relevant groupings	The crew included three white individuals and one black individual. Race is not reported in the manuscript to protect the anonymized status of the crew member IDs (C001, C002, C003, C004) given their high-profile public status.
Population characteristics	The crew member composition was of two races and ages ranged from 29-51.
Recruitment	Participants were recruited by SpaceX and mission commander Jared Isaacman through various task-specific criteria and raffle processes. There are no self-selection biases relevant to sample the collection protocols outlined in this paper.
Ethics oversight	All subjects were consented at an informed consent briefing (ICB) at SpaceX (Hawthorne, CA), and samples were collected and processed under the approval of the Institutional Review Board (IRB) at Weill Cornell Medicine, under Protocol 21-05023569. All crew members have consented for data and sample sharing.

Note that full information on the approval of the study protocol must also be provided in the manuscript.

Field-specific reporting

Please select the one below that is the best fit for your research. If you are not sure, read the appropriate sections before making your selection.

☒ Life sciences ☐ Behavioural & social sciences ☐ Ecological, evolutionary & environmental sciences

For a reference copy of the document with all sections, see nature.com/documents/nr-reporting-summary-flat.pdf

Life sciences study design

All studies must disclose on these points even when the disclosure is negative.

Sample size	The entire Inspiration4 crew was profiled, which was limited by the size of the Dragon capsule (n=4).
Data exclusions	No data has been excluded. We report all biospecimen samples collected over the course of the Inspiration4 mission.
Replication	Replication tests are difficult as mission parameters cannot be repeated. To compensate for this, the crew was profiled longitudinally, with multiple timepoints pre-flight, in-flight, and post-flight.
Randomization	This is not relevant to the study as we were profiling the entire crew longitudinally (pre-flight, in-flight, and post-flight).
Blinding	Blinding was not possible because all subjects were astronauts in the same crew.

Reporting for specific materials, systems and methods

We require information from authors about some types of materials, experimental systems and methods used in many studies. Here, indicate whether each material, system or method listed is relevant to your study. If you are not sure if a list item applies to your research, read the appropriate section before selecting a response.

Materials & experimental systems

n/a	Involved in the study	
☒	☐	Antibodies
☒	☐	Eukaryotic cell lines
☒	☐	Palaeontology and archaeology
☒	☐	Animals and other organisms
☒	☐	Clinical data
☒	☐	Dual use research of concern
☒	☐	Plants

Methods

n/a	Involved in the study	
☒	☐	ChIP-seq
☒	☐	Flow cytometry
☒	☐	MRI-based neuroimaging

Plants

Seed stocks	N/A
Novel plant genotypes	N/A
Authentication	N/A
